# Magnetoviscous Property and Hyperthermia Effect of Amorphous Nanoparticle Aqueous Ferrofluids

**DOI:** 10.1186/s11671-018-2790-0

**Published:** 2018-11-23

**Authors:** Chuncheng Yang, Mengchun Yu, Shuchun Zhao, Yuan Tian, Xiufang Bian

**Affiliations:** 1 School of Gemology and Materials Technology, Hebei GEO University, Shijiazhuang, 050031 China; 2 Key Laboratory for Liquid-Solid Structural Evolution and Processing of Materials, Ministry of Education, Shandong University, Jinan, 250061 China

**Keywords:** Amorphous nanoparticle, Aqueous ferrofluids, Viscosity, Hyperthermia effect

## Abstract

Magnetic Fe-B, Fe-Ni-B, and Co-B nanoparticles were successfully synthesized and introduced to water to prepare aqueous ferrofluids. The Fe-B, Fe-Ni-B, and Co-B particles are homogeneous amorphous nanoparticles with an average particle size 15 nm. The shape of the amorphous nanoparticles is regular. The Fe-B, Fe-Ni-B, and Co-B amorphous nanoparticles are superparamagnetic. Moreover, the saturation magnetizations of Fe-B and Fe-Ni-B amorphous nanoparticles are 75 emu/g and 51 emu/g. These are approximately 2.8 and 1.9-fold larger than Co-B nanoparticles, respectively. The viscosity of the amorphous ferrofluids has a strong response to external magnetic field. The yield stress increases with increasing magnetic field. The hyperthermia research of amorphous ferrofluids was firstly investigated. The experimental results indicate that the heating temperature of Fe-B ferrofluid and Fe-Ni-B ferrofluid could increase to 42 °C in 750 s and 960 s, respectively, when the output current is 300 A. The temperature could reach 61.6 °C for a Fe-B ferrofluid. The heating efficiencies of the amorphous ferrofluids demonstrate that the Fe-B ferrofluid and Fe-Ni-B ferrofluid may have great potential for biomedical applications.

## Introduction

Ferrofluids (FFs), also called magnetic fluids, are colloidal solutions of magnetic nanoparticles in a fluid carrier such as organic solvents, water [1–5]. As a new type of smart functional materials, FFs offer unique physical, chemical, and biocompatible properties [6–9]. FFs have been applied in biomedicine for magnetic resonance imaging (MRI) [10] and target drug delivery [11], as well as for phase separation [12], removal of water pollutants [13], and sensing [14].

The increased viscosity induced by the applied magnetic field influences FF applications. Studies on magnetoviscous properties evaluate the viscosity variations in FFs as a function of time, temperature, shear rate, or other factors under applied magnetic fields [4, 15–20]. Rajnak [18] studied the viscosity of a transformer oil-based FF and found that the electric field-induced viscosity changes are analogous to the magnetoviscous effect. Nowak [19] investigated the changing viscosity of FFs diluted with sheep blood. They found that the strong magnetoviscous effect leads to the assumption of big changes in the microstructure due to magnetic fields. Prior work demonstrated a significant interaction of the carrier medium and surfactant with a consideration of the magnetic behavior of FFs [20]. Research on the magnetoviscous properties of FFs remains a focal point. The amorphous alloys have a promising future for fuel cell electrode [21], nano-porous materials [22], biodegradation materials [23], etc. due to their unique properties related to amorphous metastable atomic structure and low-cost raw materials [24]. Other studies showed that amorphous soft magnetic Fe-based alloys have great potential applications in preparing magnetic functional fluids because of their unique magnetic properties versus crystalline alloys [25]. Fe_73.5_Nb_3_Cu_1_Si_13.5_B_9_ [26, 27] and Fe_78_Si_9_B_13_ amorphous alloy particles have been applied in magnetorheological fluids. However, it is difficult to prepare amorphous nanoparticles applied in FFs via a conventional mechanical milling method. Our group synthesized and investigated magnetic Co-Fe-Si-B [28] amorphous nanoparticles as well as Fe-Co-B [29] amorphous nanoparticles applied to FFs. These data show that the amorphous FFs exhibit good stability. Nevertheless, little attention has been given to the magnetoviscous property of FFs based on amorphous nanoparticles.

Hyperthermia therapy has been a focus of cancer treatment, and magnetic fluid hyperthermia (MFH also called FF hyperthermia) is a therapeutic procedure. FFs are injected into tissues containing cancerous cells and then exposed to a frequency alternating magnetic field, resulting in a temperature rise up to 42–45 °C to destroy the tumor cells [30–32]. Importantly, the nanoparticles in the FFs must not be toxic. Iron oxide (Fe_3_O_4_) or cobalt iron oxide (CoFe_2_O_4_) nanoparticles are popularly selected to prepare FFs for magnetic fluid hyperthermia because of their simple processing, low cost, and good biological compatibility [33–38]. Lahiri [38] studied the alternating magnetic field-induced heating of a water-based FF using infrared thermography. The FF contains tetramethyl ammonium hydroxide-coated iron oxide nanoparticles. The results indicate a higher initial rate of temperature rise and a lower maximum temperature at the end of the heating period. Zubarev [39] reported the effect of magnetic interactions between single domain ferromagnetic particles on the hyperthermia effect produced by these particles under the action of an oscillating magnetic field. However, few studies have reported hyperthermia research on amorphous magnetic nanoparticle FFs.

In this paper, magnetic Fe-B, Fe-Ni-B, and Co-B amorphous nanoparticles were successfully synthesized by a chemical reduction method. The structure, morphology, and magnetic properties of the amorphous nanoparticles were investigated. The magnetoviscous properties and hyperthermia effect of corresponding FFs were also studied. In view of the magnetic properties and prominent heating effect, the amorphous FFs as promising materials in medical applications could also offer opportunities in emerging areas such as cooling applications, energy conversion devices, printed electronics, etc.

## Materials and Methods

Ferrous sulfate (FeSO_4_•7H_2_O), cobalt chloride (CoCl_2_•6H_2_O), nickel chloride (NiCl_2_•6H_2_O), sodium borohydride (NaBH_4_), sodium hydroxide (NaOH), ethyl alcohol, agar, and polyethylene glycol (PEG-400) were used. All chemicals were of analytical reagent (AR) grade and used without further purification. Before each experiment, all glassware were cleaned with dilute nitric and repeatedly washed with deionized water.

The amorphous particles were prepared by chemical reduction. In a typical process, a solution was obtained by dissolving certain amount of FeSO_4_•7H_2_O and NiCl_2_•6H_2_O into 200 ml of 50% ethanol solution with mechanical stirring and supersonic dispersion. Then, 50 ml of 0.8 M NaBH_4_ aqueous solution was added dropwise as a reducing agent at a speed of 1.5 ml/min at 20 °C in a three-necked flask under a protective argon environment. Here, the NaOH solution was used to adjust the pH of NaBH_4_ solution to 10–12. After stirring with supersonic dispersion for 2.5 h, the black precipitate was separated using a magnet. The particles were washed with deionized water for several times. After that, appropriate 0.075 g agar was added as the first surfactant and 0.05 g PEG-400 was added as the second surfactant. These were put into the Fe-Ni-B particle suspension at a constant temperature. The mixture was stirred for 1 h at a constant temperature. Finally, the stable Fe-Ni-B amorphous aqueous FF was obtained after cooling to room temperature.

The Fe-B amorphous particles were obtained using a chemical reduction method, i.e., from the reduction of FeSO_4_•7H_2_O using NaBH_4_ as a reducing agent in aqueous solution. Co-B amorphous particles were obtained from the reduction of CoCl_2_•6H_2_O solutions. The corresponding Fe-B aqueous FF and Co-B aqueous FF were similarly obtained.

The structure and amorphous state of magnetic Fe-B, Fe-Ni-B, and Co-B amorphous nanoparticles were characterized by X-ray diffraction (XRD) measurements using a D/max-Rb, with a Ni-filtered Cu Kα radiation source. The thermal properties were characterized with a differential scanning calorimeter (Netzsch DSC 404 C) at a heating rate of 20 °C/min. The magnetic properties of the amorphous nanoparticles were measured with an alternating gradient force magnetometer (AGM) at room temperature. The morphologies of the amorphous nanoparticles were identified via transmission electron microscopy (TEM). The magnetoviscous properties of FFs were studied with a rheometer (Anton Paar MCR301) equipped with an external controllable magnetic field. The hyperthermia effects of the amorphous FFs were studied using a device shown in Fig. 8a. Field-induced heating experiments were performed using a radio frequency induction heating system (AtecD, Bamac, China) consisting of a high-frequency generator and a tank circuit equipped with water-cooled electrolytic copper coils. The experiments were performed at a fixed frequency of 90 kHz, and the magnetic field was changed by varying the coil current. An infrared thermometer (OSXL207, Omega, USA) with an accuracy of 0.1 °C was used to record the temperature in the magnetic heating experiment. The error in our temperature measurement is with 1 °C. The experimental tests were performed at room temperature.

## Results and Discussion

**Fig. 1:**
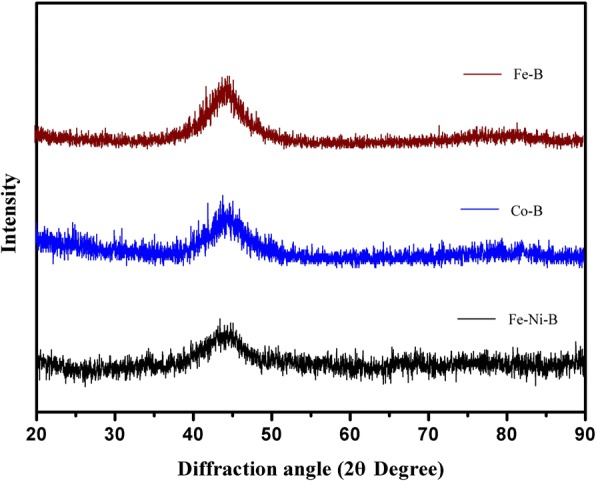
XRD patterns of Fe-B, Fe-Ni-B, and Co-B particles

Figure 1 shows the X-ray diffraction (XRD) patterns of magnetic Fe-B, Fe-Ni-B, and Co-B particles, respectively. The Fe-B, Fe-Ni-B, and Co-B particles consist of a broad single peak in the 2θ range of 40°~50° and no crystalline peak can be seen, which is characteristic of amorphous structure (Fig. 1). The results indicate that Fe-B, Fe-Ni-B, and Co-B particles have a typical amorphous structure.

**Fig. 2:**
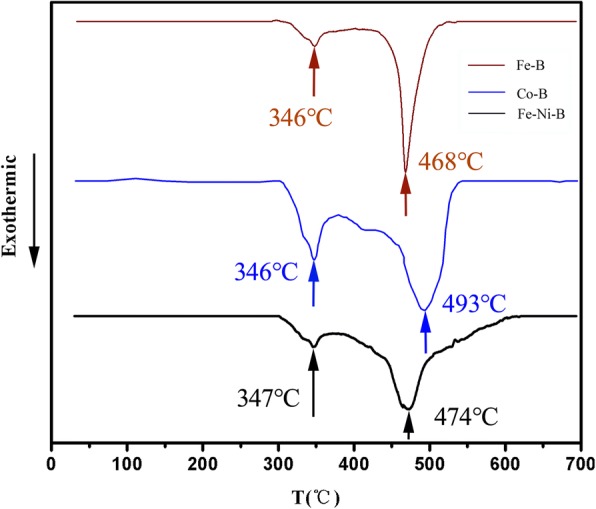
DSC curves of Fe-B, Fe-Ni-B, and Co-B particles

The differential scanning calorimeter (DSC) curves of the Fe-B, Fe-Ni-B, and Co-B particles are shown in Fig. 2. The experiments were carried out at a heating rate of 20 °C/min. Fe-B, Fe-Ni-B, and Co-B particles exhibit two exothermic peaks demonstrating two-stage crystallization processes [40]. The temperatures of two exothermic peaks are marked in Fig. 2, which could help select the annealing temperature of the amorphous particles in subsequent work. These results correspond well with the XRD data.

**Fig. 3:**
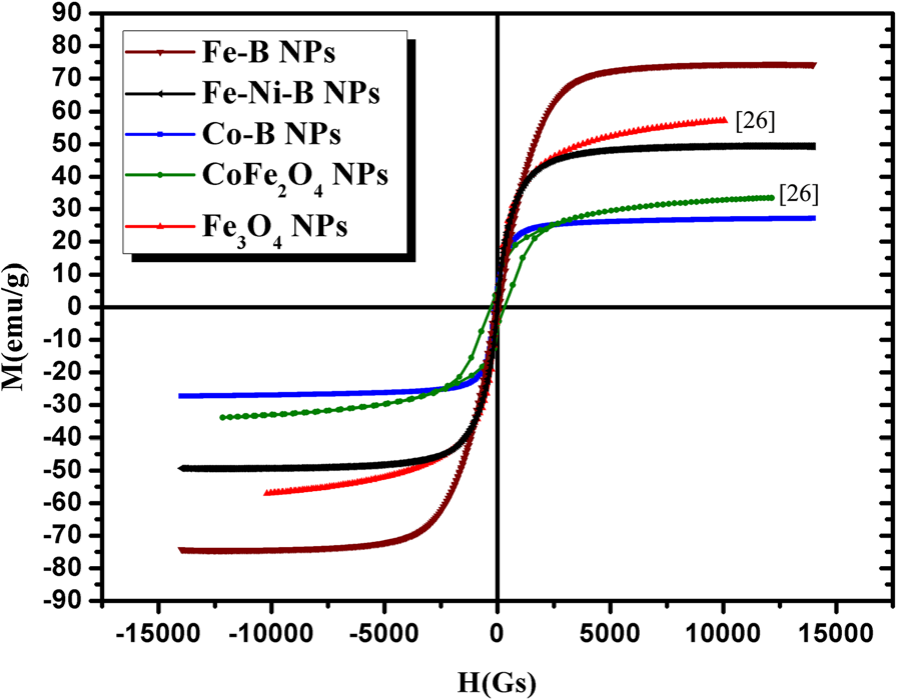
Hysteresis curves of Fe-B, Fe-Ni-B, and Co-B particles

The magnetic properties of the as-prepared Fe-B, Fe-Ni-B, and Co-B particles were characterized by AGM at room temperature. The magnetic hysteresis curves are shown in Fig. 3. The saturation magnetizations (Ms) of the Fe-B particles and Fe-Ni-B particles are 75 emu/g and 51 emu/g, respectively. Moreover, no coercivity and remanence are observed on the hysteresis curves, confirming the superparamagnetism of the F-B and Fe-Ni-B particles. The Ms of the Co-B particles is 27 emu/g; these particles also exhibit superparamagnetic behavior. In addition, the Ms of Fe-B and Fe-Ni-B particles are approximately 2.8- and 1.9-fold larger than the Co-B particles, respectively. Also we can see that the Ms of the Fe-B particles is higher than that of Fe_3_O_4_ particles and CoFe_2_O_4_ particles [26]. The structure, size, magnetization, and concentration of different FF samples can be seen in Table 1.

**Table 1:** The structure, size, magnetization, and concentration of different FF samples

FFs composition	Structure	Physical diameter by TEM (nm)	Magnetization at 14 kOe (emu/g)	Concentration (wt%)
Fe-B	Amorphous	11–14	75	1.8
Fe-Ni-B	Amorphous	12–15	51	1.8
Co-B	Amorphous	13–15	27	1.8

**Fig. 4:**
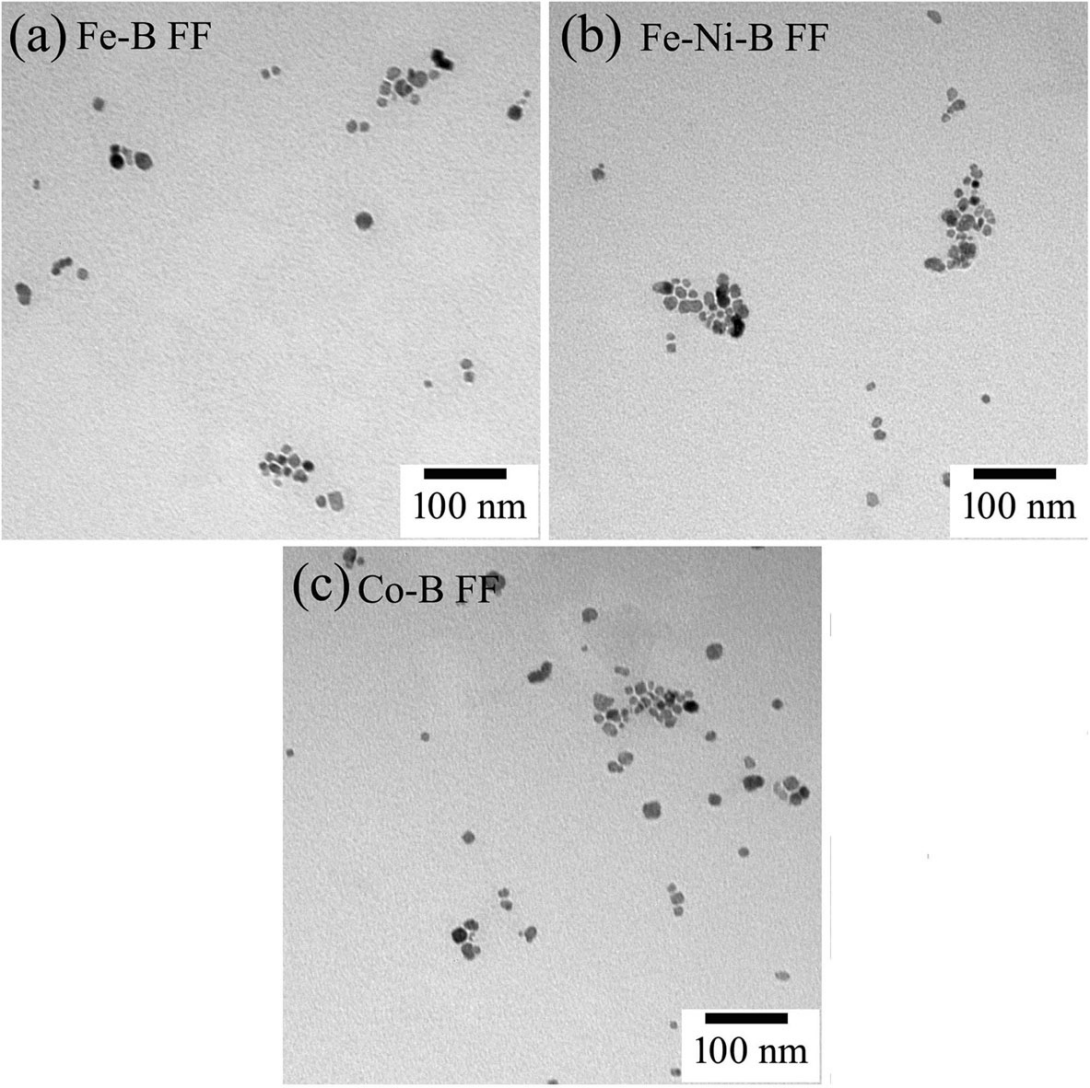
TEM images of Fe-B FF (**a**), Fe-Ni-B FF (**b**), and Co-B FF (**c**)

We next investigated the morphologies of the amorphous particles in FFs with TEM (Fig. 4). The FFs were diluted and then dispersed in an ultrasonic for 20 min. The support films adhered with a copper net were immersed in diluted FFs. The specimens were prepared well after drying the sample in an oven for 30 min. The TEM images shown in Fig. 4 demonstrate that the amorphous particles in FFs are nearly spherical. The average mean diameters of the amorphous particles are ~ 15 nm.

**Fig. 5:**
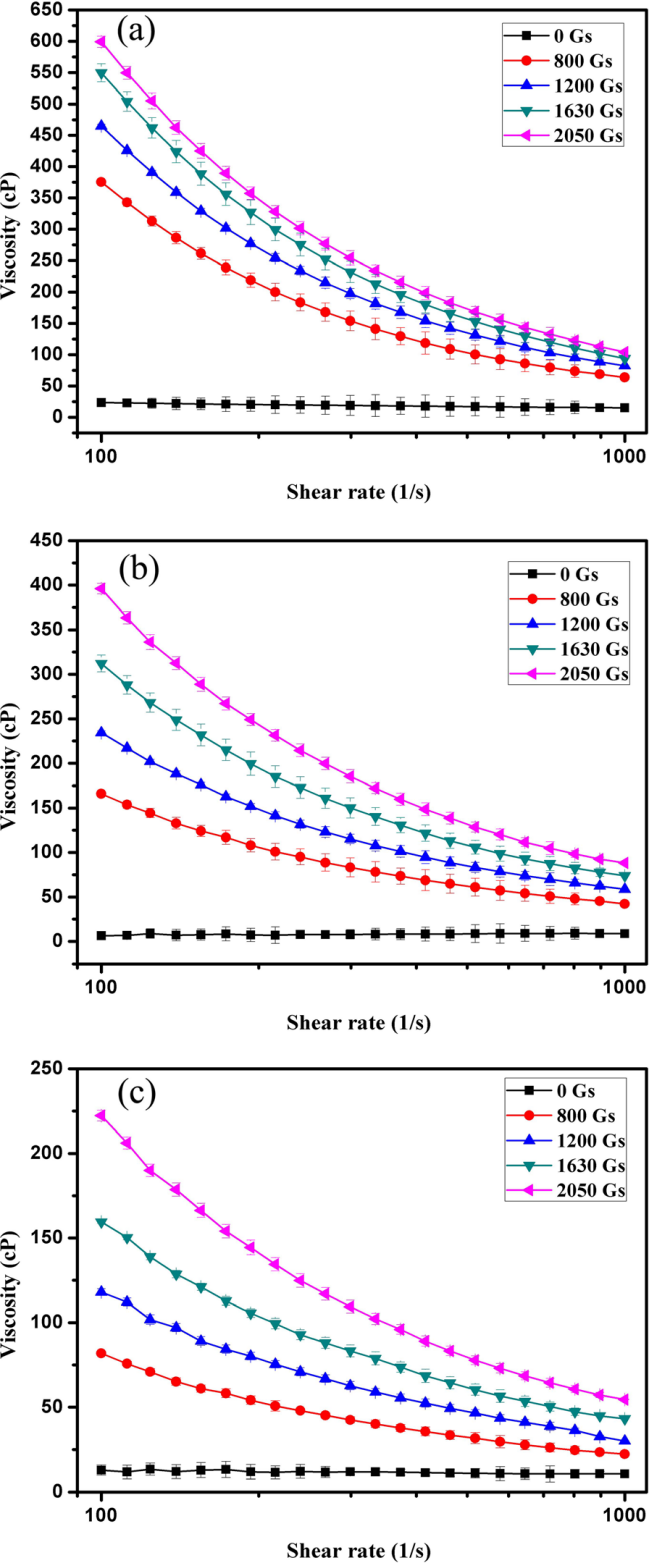
The viscosity as a function of shear rate for Fe-B FF (**a**), Fe-Ni-B FF (**b**), and Co-B FF(**c**)

The magnetoviscous properties of the three amorphous FFs (Fe-B FF, Fe-Ni-B FF, and Co-B FF) with 1.8 wt% of magnetic particles were investigated by a rheometer with an external controllable magnetic field. The viscosity of each sample was measured two times at a constant set temperature 25 °C. Every time the sample went through one cycle of shear rate sweep ramping up from 100 to 1000 1/s and then ramping down from 1000 to 100 1/s. The average value was obtained by calculating the viscosity at the same shear rate. The viscosity-shear rate curves of amorphous FFs under different external magnetic fields on a logarithmic scale are shown in Fig. 5. All the amorphous FFs (Fe-B FF in Fig. 5a, Fe-Ni-B FF in Fig. 5b, and Co-B FF in Fig. 5c) exhibit shear shining behavior under different magnetic fields. The viscosity decreases with increasing shear rates. The Fe-B FF has a larger viscosity than Fe-Ni-B FF and Co-B FF. This is because of the Ms of the amorphous Fe-B nanoparticles, Fe-Ni-B nanoparticles, and Co-B nanoparticles.

**Fig. 6:**
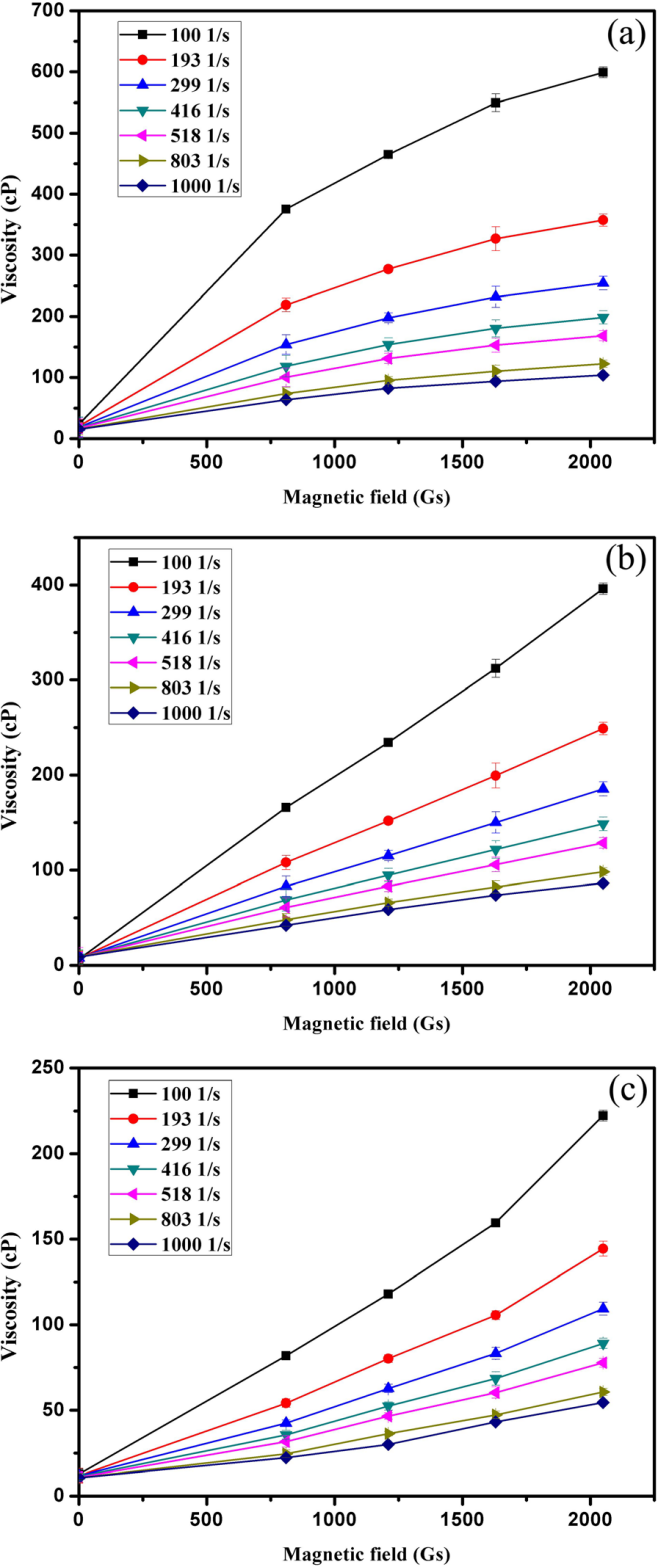
The viscosity as a function of magnetic field for Fe-B FF (**a**), Fe-Ni-B FF (**b**), and Co-B FF(**c**)

The magnetic field also plays an important role in the viscosity of amorphous FFs. The viscosity is shown as a function of magnetic field in Fig. 6. The results demonstrate that the viscosity of all amorphous FFs increase with increasing external magnetic field. This corresponds well with the results in Fig. 5. The magnetic amorphous nanoparticles in FFs rearranged their orientation when a magnetic field was applied. It aligned in the direction of magnetic field. The interaction and arrangement of the nanoparticles in the FFs became stronger with increasing magnetic field intensity, which led to increased flow resistance. Moreover, prior reports [15, 41–46] show that with increasing magnetic field, chain-like or drop-like structures, and aggregation could form in FFs, which leads to a remarkable increase in viscosity. The observed shear thinning behavior in Fig. 5 could be explained by breaking of these chains or drops due to shear. The nanoparticles begin to arrange their orientation in the shearing direction when the applied shear rate increases. Moreover, the increasing shear rate destroys chains or drop-like aggregates; consequently, the FF viscosity decreases.

**Fig. 7:**
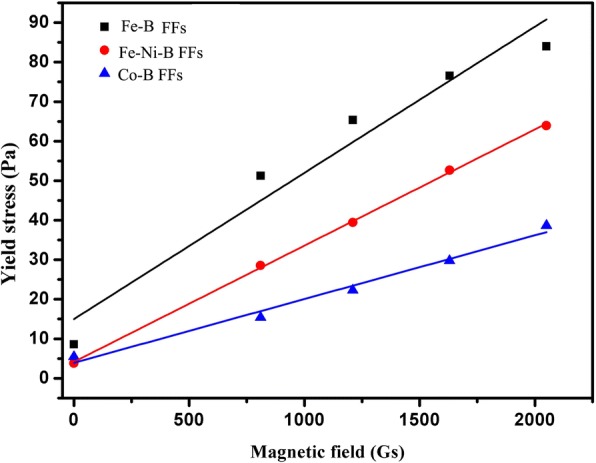
The yield stress as a function of magnetic field for Fe-B FF, Fe-Ni-B FF, and Co-B FF

The yield stress of FF can be obtained via linear extrapolation, and the intercept of each fitting curve is considered to be the yield stress of the FF under the corresponding magnetic field [27]. Therefore, the yield stresses of the three amorphous FFs under different magnetic fields are obtained in Fig. 7. It demonstrates that the yield stress of FFs increases with increasing magnetic strength especially for the amorphous Fe-B FF. This is because chain-like or drop-like structures as well as aggregates are formed under the applied magnetic field. The force between amorphous nanoparticles becomes stronger while increasing magnetic strength. Prior work [47] showed that the yield stress of amorphous FFs is due to the magnetization of the magnetic amorphous nanoparticles.

**Fig. 8:**
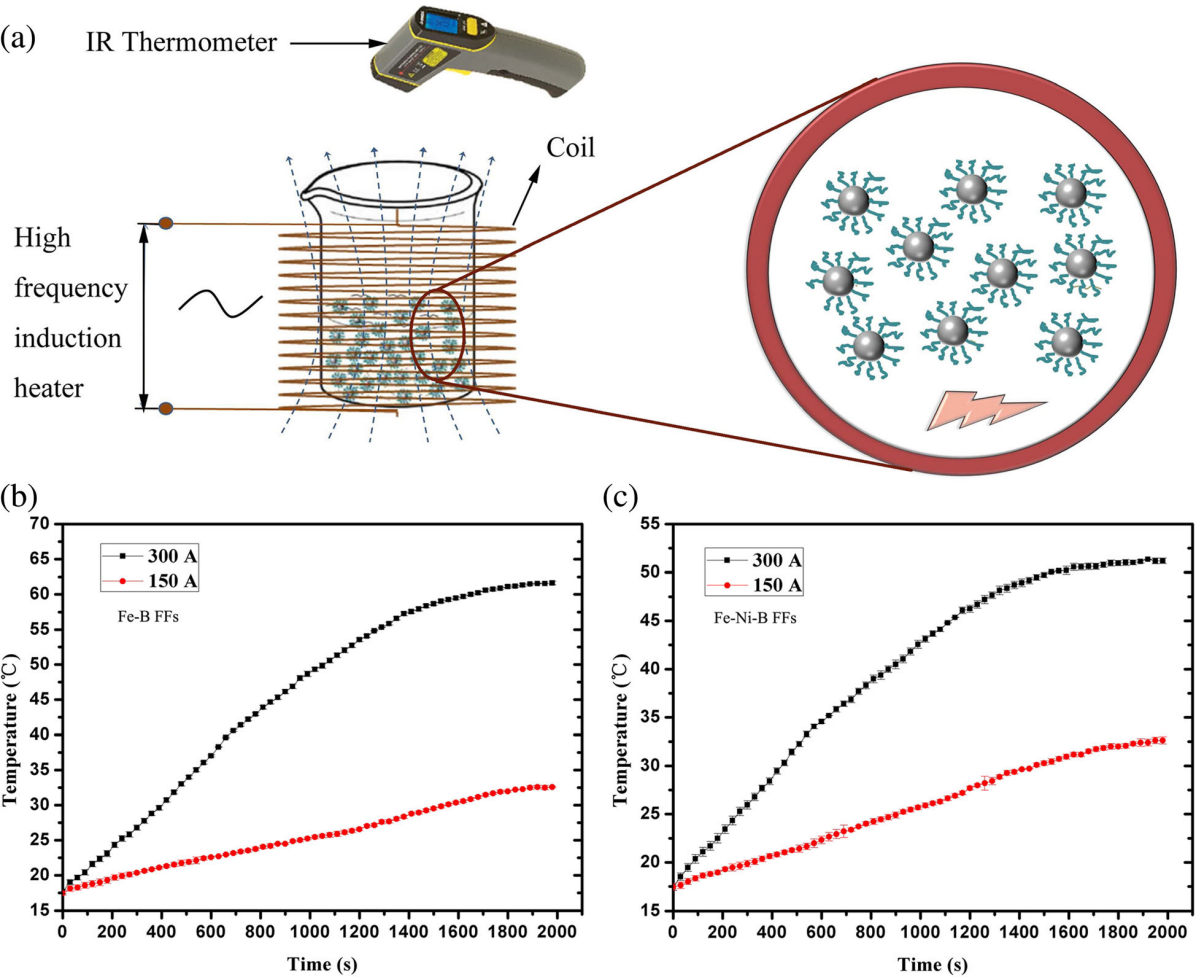
The schematic map of experimental setup for the magnetic heating experiment (**a**), the heating curves of the amorphous Fe-B FF (**b**), and the heating curves of the amorphous Fe-Ni-B FF(**c**)

FF hyperthermia has attached much importance due to its safety and limited physical or mental strain on the patients [26, 48–50]. Such hyperthermia is induced by heating effects in an alternating current (AC) magnetic field. We studied the hyperthermia effects of FFs with Fe-based amorphous nanoparticles, i.e., Fe-B FF and Fe-Ni-B FF. A schematic map of the experimental device is shown in Fig. 8a. An IR thermometer with an accuracy of 0.1 °C recorded the temperature in the magnetic heating experiment. The error in our temperature measurement is 1 °C. The tests were carried out at room temperature. The magnetic heating experiments were conducted by changing variable output currents ranging from 150 to 300 A. Then, 50 ml Fe-B FF and Fe-Ni-B FF at 5 wt% were studied. The experimental conditions are as described previously [26]. The work frequency of the induction heater in our experiment was 90 kHz. The work frequency is 50–100 kHz, which is safe for biomedical applications [51].

The magnetic heating results are shown in Fig. 8b, c. The temperatures of both Fe-B FF in Fig. 8b and Fe-Ni-B FF in Fig. 8c increased markedly with time. The temperature increased with increasing electrical output currents. The temperatures of the FFs under different output currents were recorded at 2000 s (in Table 2). When the electrical output current was controlled at 150 A, the temperature could rise to 32.5 °C for Fe-B FF and to 32.6 °C for Fe-Ni-B FF. When the output current was 300 A, the final stable temperature was 61.6 °C and 51.2 °C for Fe-B FF and Fe-Ni-B FF, respectively. The heating efficiency of the hyperthermia effect of Fe-B FF is about 20.3% higher than that of Fe-Ni-B FF (Table 2). The hyperthermia results indicate that when the electrical current was controlled at 300 A, the temperature of Fe-B FF and Fe-Ni-B FF could raise to 42 °C in 750 s and 960 s, respectively. The specific absorption rates (SARs) could be calculated from the field assisted heating curves [52, 53]. The specific heat capacity and density of water in our paper were considered as 4.18 J g^−1^ K^−1^ and 1 g/cc, respectively. The SAR values were 21.91 W/g for Fe-B FF and 19.48 W/g for Fe-Ni-B FF, respectively. The SAR values were 76.15 W/g and 69.97 W/g for Fe-B FF and Fe-Ni-B FF, respectively, when the output current was 300 A. The heating experiments demonstrate that the intensity of alternating magnetic fields induced by electrical currents affect the hyperthermia of the amorphous FFs. The heating could be controlled effectively by adjusting the output current.

**Table. 2:** The temperatures of FFs in 2000 s under different output currents

FFs	T-150 A (°C)	T-300 A (°C)
Fe-B amorphous FF	32.5	61.6
Fe-Ni-B amorphous FF	32.6	51.2
(T_Fe-B_-T_Fe-Ni-B_)/T_Fe-Ni-B_	–	20.3%

The heating effects of aqueous FFs are mainly attributed to Neel relaxation (magnetic dipole rotates within the particle) and Brownian relaxation mechanism (particle rotation against the hydrodynamic resistance of the carrier fluid) [54–56]. Based on the domain theory, the critical diameters of single domain are 19.6 nm, 19.2 nm, and 42.4 nm for Fe, Co, and Ni nanoparticles, respectively [57]. Here, we assume that the Fe-B amorphous nanoparticles and Fe-Ni-B amorphous nanoparticles should possess single domain structures. The magnetic spins align randomly under no external fields due to the thermal energy. When an AC field is applied, the single domain changes its magnetization orientation in response to the AC fields, and the magnetic energy is simultaneously converted into thermal energy. We conclude that the Fe-B amorphous FF and Fe-Ni-B amorphous FF have significant heating effects suggesting that Fe-B amorphous FF and Fe-Ni-B amorphous FF have a promising future for hyperthermia treatment.

## Conclusions

Magnetic Fe-B, Fe-Ni-B, and Co-B amorphous nanoparticles as well as the corresponding amorphous FFs were successfully synthesized. The nanoparticles are homogenous with amorphous structures. The shape of the amorphous particles is regular. The Fe-B, Fe-Ni-B, and Co-B amorphous nanoparticles show superparamagnetic. The Ms of Fe-B and Fe-Ni-B amorphous nanoparticles are 75 emu/g and 51 emu/g. This is approximately 2.8 and 1.9 times larger than Co-B nanoparticles, respectively. The amorphous FFs have a strong response to an external magnetic field. The yield stress increases with increasing magnetic field. The hyperthermia results indicate that when alternating electrical output current is controlled at 300 A, the temperature of Fe-B FFs and Fe-Ni-B FFs could rise to 42 °C in 750 s and 960 s, respectively. The final stable temperature was 62 °C for Fe-B FFs. The heating efficiencies of amorphous FFs demonstrate that Fe-based amorphous FFs have great potential for biomedical applications. Indeed, studies on the magnetoviscous properties of amorphous FFs and the mechanism of hyperthermia effect for amorphous FFs remains unclear and will stimulate future work.

## Abbreviations

AGM: Alternating gradient force magnetometer
DSC: Differential scanning calorimeter
FFs: Ferrofluids
MFH: Magnetic fluid hyperthermia
Ms: Saturation magnetization
SAR: Specific absorption rate
TEM: Transmission electron microscopy
XRD: X-ray diffraction
